# Clinical validity of biochemical and molecular analysis in diagnosing Leigh syndrome: a study of 106 Japanese patients

**DOI:** 10.1007/s10545-017-0042-6

**Published:** 2017-04-20

**Authors:** Erika Ogawa, Masaru Shimura, Takuya Fushimi, Makiko Tajika, Keiko Ichimoto, Ayako Matsunaga, Tomoko Tsuruoka, Mika Ishige, Tatsuo Fuchigami, Taro Yamazaki, Masato Mori, Masakazu Kohda, Yoshihito Kishita, Yasushi Okazaki, Shori Takahashi, Akira Ohtake, Kei Murayama

**Affiliations:** 1 Department of Metabolism, Chiba Children’s Hospital, 579-1 Heta-cho, Midori-ku, Chiba 266-0007 Japan; 2 Department of Pediatrics and Child Health, Nihon University School of Medicine, 30-1 Ohyaguchikami-cho, Itabashi-ku, Tokyo, 173-8610 Japan; 3 Department of Pediatrics, Saitama Medical University, 38 Morohongo, Moroyama, Saitama 350-0495 Japan; 4 Department of Pediatrics, Matsudo City Hospital, Matsudo, 4005 Kamihongo, Matsudo, Chiba 271-8511 Japan; 5 Division of Translational Research, Research Center for Genomic Medicine, Saitama Medical University, 1397-1 Yamane, Hidaka, Saitama, 350-1241 Japan; 6 Division of Functional Genomics and Systems Medicine, Research Center for Genomic Medicine, Saitama Medical University, 1397-1 Yamane, Hidaka, Saitama 350-1241 Japan

**Keywords:** Mitochondrial respiratory chain disorder, Leigh syndrome, Enzyme assay, Genetic analysis, Oxygen consumption rate

## Abstract

Leigh syndrome (LS) is a progressive neurodegenerative disorder of infancy and early childhood. It is clinically diagnosed by typical manifestations and characteristic computed tomography (CT) or magnetic resonance imaging (MRI) studies. Unravelling mitochondrial respiratory chain (MRC) dysfunction behind LS is essential for deeper understanding of the disease, which may lead to the development of new therapies and cure. The aim of this study was to evaluate the clinical validity of various diagnostic tools in confirming MRC disorder in LS and Leigh-like syndrome (LL). The results of enzyme assays, molecular analysis, and cellular oxygen consumption rate (OCR) measurements were examined. Of 106 patients, 41 were biochemically and genetically verified, and 34 had reduced MRC activity but no causative mutations. Seven patients with normal MRC complex activities had mutations in the *MT-ATP6* gene. Five further patients with normal activity in MRC were identified with causative mutations. Conversely, 12 out of 60 enzyme assays performed for genetically verified patients returned normal results. No biochemical or genetic background was confirmed for 19 patients. OCR was reduced in ten out of 19 patients with negative enzyme assay results. Inconsistent enzyme assay results between fibroblast and skeletal muscle biopsy samples were observed in 33% of 37 simultaneously analyzed cases. These data suggest that highest diagnostic rate is reached using a combined enzymatic and genetic approach, analyzing more than one type of biological materials where suitable. Microscale oxygraphy detected MRC impairment in 50% cases with no defect in MRC complex activities.

**Electronic supplementary material:**

The online version of this article (doi:10.1007/s10545-017-0042-6) contains supplementary material, which is available to authorized users.

## Introduction

Leigh syndrome (LS) (OMIM 256000), also known as subacute necrotizing encephalopathy, is a progressive neurodegenerative disorder associated with primary or secondary dysfunction of mitochondrial oxidative phosphorylation. Clinical manifestations include psychomotor regression or retardation and signs of brainstem dysfunction, such as respiratory disturbance, nystagmus, ophthalmoplegia, or dysphagia (Thorburn and Rahman 1993). Symptoms often start in infancy, and many patients do not survive into childhood (Sofou et al. 2014). LS was originally defined neuropathologically by bilateral necrotic lesions in the basal ganglia and/or brainstem that were found at autopsy (Leigh 1951). Such lesions can now be observed in vivo with brain magnetic resonance imaging (MRI) or computed tomography (CT) (Gropman 2013). LS is clinically diagnosed based on typical manifestations and neuroimaging, accompanied by an elevated lactate or lactate-to-pyruvate (L/P) ratio in the blood or cerebrospinal fluid (CSF). The clinical diagnosis is followed by enzyme assays and genetic analysis to confirm the biochemical and molecular background (Baertling et al. 2014).

With advances in biochemical techniques and genomic medicine, enzyme assays and genetic analyses are now standard procedures for confirming mitochondrial respiratory chain (MRC) disorders. Numerous reports on the biochemical and molecular profiles of LS have been published, but there are limited studies on clinically diagnosed LS with negative biochemical or molecular findings (Sofou et al. 2014), and the clinical validity of these diagnostic methods remains unknown. In this report, we present the results of 106 Japanese patients with LS and Leigh-like syndrome (LL) to evaluate the clinical validity of various diagnostic methods. We also assessed the detection rate of each type of biological material for the enzyme assays to determine which was optimal for diagnosing LS/LL patients. We also assessed the usefulness of microscale oxygraphy.

## Patients and methods

### Patients

A total of 106 patients were included in this study. Patients were referred to either Chiba Children’s Hospital or Saitama Medical University for enzyme assay and genetic analysis of MRC disorders from February 2007 to February 2015 by pediatricians and neurologists across Japan. Written informed consent was obtained from the parents of each patient. Both institutions received approval for comprehensive MRC analysis and genetic analysis from their appropriate ethics review boards. Data on the present illness, laboratory results, and neuroimaging findings were extracted from case summaries that accompanied the samples.

We used the stringent criteria defined by Rahman as the inclusion criteria for LS (Rahman et al. 1996). Those with atypical or normal neuroimaging results, or those with typical neuroimaging but with normal lactate levels in serum and CSF were classified as LL patients (Rahman et al. 1996). Patients were excluded from the study when they were diagnosed with pyruvate dehydrogenase complex deficiency or eventually diagnosed as having other metabolic diseases.

### Measurements

Activities of MRC complexes I, II, III, and IV were assayed in mitochondria isolated from skin fibroblasts or in the crude supernatant following centrifugation at 600 g from tissues, as previously described (Kirby et al. 1999; Murayama et al. 2009). Enzyme activities of each complex were presented as the percentage of normal control mean relative to appropriate reference enzyme activities, such as citrate synthase or MRC complex II. Enzyme activity was defined as being decreased at <40% in a cell line or <30% in a tissue, as reported (Bernier et al. 2002).

The cellular oxygen consumption rate (OCR) of fibroblast-derived cell lines was measured using microscale oxygraphy (Seahorse XF96 system; Seahorse Bioscience, Billerica, MA, USA) in cases with negative enzyme assay results. Material was prepared as reported (Kopajtich et al. 2014). After measurement of the basal OCR, oligomycin, carbonyl cyanide phenylhydrazone, and rotenone were added sequentially, and OCR was recorded after each addition. Maximum respiration rate (MRR) corresponds to the OCR after the addition of carbonyl cyanide phenylhydrazone minus rotenone-insensitive OCR (Invernizzi et al. 2012). Samples were measured in a 96-well plate, using 16 wells for each sample. Each sample’s data were normalized as 20,000 cells per well. We analyzed five control samples, each one being measured at least five times. Cells in passages five through nine were used for controls and patient samples. In each run, we measured one or two controls with patient samples. OCR was expressed as percentage relative to the average of control(s).

Patients with MRC defects by enzyme assay were analyzed for mitochondrial DNA (mtDNA) mutations by whole mtDNA sequencing. Where no causative mtDNA mutations were found, we proceeded to whole-exome sequencing with next-generation sequencing for nuclear DNA (nDNA) mutations. Detailed information on this procedure was previously reported (Kohda et al. 2016). Those with negative enzyme assay results were screened for mutations using targeted gene panel of 251 nuclear genes known to cause mitochondrial diseases as well as the whole mitochondrial genome. In a few cases where referring clinicians had screened for and identified common mtDNA mutations before referring patients to our institutions, findings were negative in our enzyme assay. There was also one case in whom an outside laboratory identified an nDNA mutation, although it was biochemically negative in our assay. The results of these cases were incorporated into the study to estimate the detection rate of each diagnostic method.

### Statistical analysis

Statistical analysis was performed using Microsoft Excel 2010 (Microsoft, Redmond, WA, USA). The Kruskal–Wallis* H* test was used to evaluate differences in continuous variables between groups, chi-squared and Fisher’s tests were used to evaluate differences between categorical variables, and Wilcoxon test was used to evaluate differences between control and patient samples. All statistical tests were two sided, and *p* values <0.05 were considered statistically significant.

## Results

### Overview

All 106 analyzed patients were from different families, and no consanguinity was reported. Seventy-five patients showed MRC defects that satisfied Bernier’s criteria (Table 1). Forty-one of those patients received a molecular diagnosis: nDNA mutations in 19 and mtDNA mutations in 22. In 34 patients, the underlying genetic mutation was not identified. Of the 31 patients with no apparent reduction in MRC activities, seven had mutations in *MT-ATP6*, one in *MT-ND6*, three in *ECHS1*, and one in *SLC19A3*. The remaining 19 patients had no biochemical defect in MRC and no confirmed genetic diagnosis, including two patients whose gene analysis was not performed due to lack of material. Microscale oxygraphy was performed in 19 available fibroblast cell lines, with no reduction in enzyme activities and a significant reduction in OCR observed in ten.

**Table 1:** Mitochondrial respiratory chain (MRC) complex activities and associated genetic mutations

Mutation	Complex I–IV activity (enzyme assay)		Total
	Decreased	Not decreased	
nDNA	19	4	23
mtDNA	22	8	30
None confirmed	34	19	53
Total	75	31	106

*nDNA* nuclear DNA, *mtDNA* mitochondrial DNA

### Clinical presentation

Patient clinical features and metabolic status are summarized in Table 2 according to their biochemical and genetic backgrounds:

1. Positive assay and mutation identified (41 patients)
2. Mutation only (12 patients)
3. Positive assay only (34 patients)
4. Negative assay and no confirmed genetic diagnosis (19 patients).

There was no apparent clinical difference between groups. Patient status, age of living patients, LS/LL ratio, and median age at onset were similar. Besides regression and developmental delay, seizure and respiratory distress were the two major clinical symptoms observed in each group. There were no differences in serum or CSF lactate levels between groups. The mean serum and CSF L/P ratios for the whole cohort were 23.0 ± 13.2 and 24.1 ± 18.9, respectively, which were higher than the L/P ratios in normal individuals (Saudubray and Charpentier 2001), with no significant difference between groups.

**Table 2:** Clinical presentations of patients with Leigh syndrome

	Defect and mut	Mut only	Defect only	No defect, no validated mut	Total
Number of patients	41	12	34	19	106
Leigh-like	6	4	10	4	24
Living^a^	71% (20/28)	78% (7/9)	62% (16/26)	83% (10/12)	71% (53/75)
Age of living patients^a^ [median (range)]	9 (3–17) years	8 (3–15) years	9.5 (3–38) years	8.5 (6–20) years	8 (3–38) years
Age at onset [median (range)]	10.5 months (0 months–8 years)	9 months (0 months–5 years)	5.5 months (0 months–6 years)	10 months (0 months–2 years)	9 months (0 months–8 years)
Neonatal onset	2 (5%)	2 (17%)	7 (21%)	1 (5%)	12 (11%)
Seizure	20%	33%	41%	42%	32%
Involuntary movement	10%	25%	18%	16%	15%
Hypotonia	24%	42%	9%	32%	23%
Nystagmus/ ophthalmoplegia	17%	33%	26%	11%	21%
Dysphagia	10%	17%	29%	32%	21%
Respiratory distress	24%	17%	41%	37%	31%
Serum L/P (mean ± SD) (number of data available)	26.4 ± 16.4 (36)	22.9 ± 14.9 (10)	21.4 ± 9.9 (27)	18.2 ± 5.7 (16)	23.0 ± 13.2 (89)
CSF L/P (mean ± SD) (number of data available)	27.2 ± 28.0 (30)	20.7 ± 4.5 (9)	25.0 ± 9.6 (20)	18.7 ± 7.1 (14)	24.1 ± 18.9 (73)

*Mut* mutations in mitochondrial and nuclear DNA, *L/P* lactate-to-pyruvate ratio,* SD* standard deviation, *CSF* cerebrospinal fluid

^a^As of November 2016

### Enzyme assay

A total of 154 samples (92 fibroblast, 56 skeletal muscle, four liver, one cardiac muscle, and one lymph node) were submitted for enzyme assay, and a total of 151 assays (91 fibroblasts, 55 skeletal muscle, four liver, and one cardiac muscle sample) were completed. Of these, 89 assays (59%) exhibited decreased activity: fibroblasts, 54/91 (59%); skeletal muscle, 31/55 (56%); liver, 4/4 (100%); and cardiac muscle, 0/1 (0%), confirming MRC disorder in 75 (71%) of the 106 patients analyzed. No significant difference was found between the detection rate of fibroblasts and skeletal muscle biopsy samples. Isolated complex I defect was most frequently observed (37 patients), followed by isolated complex IV (17). Combined complex defects were observed in 20 patients, and the most frequently observed combination was defects of complexes I and IV (13).

In 42 patients, more than one type of tissue material was assayed; results were inconsistent in 17. Excluding those with mutations in the *MT-ATP6* gene, 37 patients had both skeletal muscle biopsy samples and fibroblasts assayed; results were inconsistent in 13 (Supplementary Table 1). Inconsistency was observed in four patients with nDNA mutations, in one with mtDNA mutation, and in eight with no genetic background confirmed. For genetically verified patients excluding those with mutations in the *MT-ATP6* gene, 60 samples were analyzed by enzyme assay; 12 returned normal or nonsignificant results, the majority of which were from patients with nDNA mutations (Supplementary Table 2).

### Oxygen consumption rate

The OCR was measured in 19 of the 31 LS/LL patients who presented normal enzyme assay results. Seven cases with mtDNA mutations were omitted. Analysis was precluded in three cases from whom fibroblast cell lines were not available. In an additional two patients, cell lines did not react properly to the experiment, and results were not obtained. Based on MRR distribution in our five controls, a reduction to <71.6% was considered a significant decline (*p* < 0.05). In 19 patients, it ranged from 36% to 136%, with a median of 69% of normal control(s). Ten patients showed a significant decline, suggesting mitochondrial respiratory dysfunction (Table 3).

**Table 3:** Oxygen consumption rate (OCR) measured with a Seahorse analyzer

Patient	Enzyme analysis	MRR (%)
Pt139	ns (Fb)	136
Pt156	ns (Fb)	**69**
Pt161	ns (Fb)	**36**
Pt207	ns (M, Fb)	90
Pt216	ns (M, Fb)	94
Pt394	ns (Fb)	94
Pt430	ns (M, Fb)	**62**
Pt536	ns (M, Fb)	96
Pt545	ns (M, Fb)	**53**
Pt668	ns (M, Fb)	**62**
Pt696	ns (Fb)	127
Pt701	ns (Fb)	**61**
Pt703	ns (M, Fb)	81
Pt794	ns (Fb)	**48**
Pt822	ns (M, Fb)	108
Pt840	ns (Fb)	**43**
Pt1038	ns (Fb)	**51**
Pt1065	ns (M, Fb)	78
Pt1120	ns (Fb)	**51**

MRR reduction to <71.6% of normal control value was considered to indicate mitochondrial impairment and is shown in bold

*OCR* oxygen consumption rate, *MRR* maximum respiration rate, *ns* not significant, *M* skeletal muscle, *Fb* cultured fibroblast, *CIV* complex IV, *P* partial decline

### mtDNA analysis

Analysis of mtDNA mutation was performed for 103 patients and were identified in 30 patients across seven different genes (Table 4), resulting in a yield of 29%. *MT-ATP6* was the gene most frequent (ten patients). We also identified 19 patients with 11 different mutations in mtDNA genes related to complex I.

**Table 4:** Mutations in mitochondrial DNA (mtDNA) and nuclear DNA (nDNA)

Patient	Gene	Mutation	LS/LL	Enzyme assay	Heteroplasmy rate (%)	Tissue
Pt27	*SURF1* (NM_003172.2)	c.743C>A:p.A248D	LS	CIV		
		c.743C>A:p.A248D				
Pt756	*SURF1* (NM_003172.2)	c.367_368del:p.R123Gfs	LS	CIV		
		c.54+1G>T				
Pt981	*SURF1* (NM_003172.2)	c.743C>A:p.A248D	LL	CIV		
		c.54+1G>T				
Pt1066	*SURF1* (NM_003172.2)	c.367_368del:p.R123Gfs	LS	CIV		
		c.867G>A:p.W289X				
Pt1143	*SURF1* (NM_003172.2)	c.743C>A:p.A248D	LS	CIV		
		c.826_827ins18:p.V276_T277ins6				
Pt312^a^	*NDUFA1* (NM_004541)	c.55C>T:p.P19S	LS	CI		
Pt286	*BOLA3* (NM_212552)	c.287A>G:p.H96R	LS	CC (I, II)		
		c.287A>G:p.H96R				
Pt376	*ECHS1* (NM_004092)	c.98T>C:p.F33S	LS	CIV		
		c.176A>G:p.N59S				
Pt536	*ECHS1* (ENST00000368547)	c.5C>T:p.A2V	LS	ns		
		c.1A>G:p.M1V				
Pt1038	*ECHS1* (NM_004092)	c.5C>T:p.A2V	LS	ns		
		c.176A>G:p.N59S				
Pt1135	*ECHS1* (NM_004092)	c.5C>T:p.A2V	LS	CI		
		c.176A>G:p.N59S				
Pt101	*NDUFAF6* (NM_152416)	c.371T>C:p.I124T	LS	CI		
		c.805C>G:p.H269D				
Pt330	*NDUFAF6* (NM_152416)	c.820A>G:p.R274G	LS	CI		
		c.820A>G:p.R274G				
Pt512	*NDUFAF6* (NM_152416)	c.226T>C:p.S76P	LS	CI		
		c.805C>G:p.H269D				
Pt598	*NDUFAF6* (NM_152416)	c.206A>T:p.D69V	LL	CI		
		c.371T>C:p.I124T				
Pt866	*NDUFAF6* (NM_152416)	c.371T>C:p.1124T	LS	CI		
		c.805C>G:p.H269D				
Pt711	*NDUFS4* (NM_002495)	c.340T>C:p.W114R	LS	CI		
		c.340T>C:p.W114R				
Pt1087	*NDUFS6* (NM_004553)	c.309+5G>A	LS	CC (I, IV)		
		c.343T>C:p.C115R				
Pt1177	*NDUFV2* (NM_021074)	c.427C>T:p.R143X	LS	CI		
		c.580G>A:p.E194K				
Pt628	*SCO2* (NM_001169109)	c.577G>A:p.G193S	LS	CC (I, IV)		
		c.773T>C:p.M258T				
Pt751	*GTPBP3* (NM_032620)	c.8G>T:p.R3L	LS	CC (I, IV)		
		c.923_947del:p.E309Rfs				
Pt156	*SLC19A3* (NM_025243)	c.372C>G:p.Y124X	LS	ns		
		c.265A>C:p.S89R				
Pt416	*MT-ND1*	m.3697G>A:p.G131S	LS	CI	100	F
Pt619	*MT-ND1*	m.3946G>A:p.E214K	LS	CC (I, IV)	66	M
Pt179	*MT-ATP6*	m.8993T>G:pL156R	LL	ns	nearly 100	B
Pt274	*MT-ATP6*	m.8993T>C:p.L156P	LS	CC (I, III)	100	F
Pt453	*MT-ATP6*	m.8993T>G:p.L156R	LS	CC (I, IV)	100	F
Pt341	*MT-ATP6*	m.8993T>C:p.L156R	LS	ns	100	M
Pt720	*MT-ATP6*	m.8993T>G:p.L156R	LS	ns	nearly 100	B
Pt772	*MT-ATP6*	m.8993T>G:p.L156R	LS	ns	nearly 100	M
Pt968	*MT-ATP6*	m.8993T>G:p.L156R	LS	ns	nearly 100	B
Pt400	*MT-ATP6*	m.9176T>C:p.L217P	LS	ns	100	B
Pt698	*MT-ATP6*	m.9176T>C:p.L217P	LS	CIV	100	B
Pt127	*MT-ATP6*	m.9185T>C:p.L220P	LL	ns	80	B
Pt728	*MT-ND3*	m.10158T>C:p.S34P	LS	CI	80	B
Pt994	*MT-ND3*	m.10158T>C:p.S34P	LS	CI	100	B
Pt43	*MT-ND3*	m.10191T>C:pS45P	LS	CI	100	F
Pt44	*MT-ND3*	m.10191T>C:pS45P	LS	CI	69	F
Pt58	*MT-ND3*	m.10191T>C:pS45P	LS	CI	na	
Pt83	*MT-ND3*	m.10191T>C:pS45P	LS	CI	100	F
Pt108	*MT-ND3*	m.10191T>C:p.S45P	LS	CI	95	B
Pt965	*MT-ND3*	m.10197G>C:pA47P (VUS)^b^	LL	CC(I,III,IV)	na	
Pt190	*MT-ND4*	m.11246G>A:pA163T (VUS)	LS	CC (I, IV)	73	F
Pt153	*MT-ND5*	m.13094T>C:pV253A	LS	CC (I, IV)	na	B,M
Pt467	*MT-ND5*	m.13513G>A:p.D393N	LL	CI	59	B
Pt744	*MT-ND5*	m.13513G>A:p.D393N	LL	CC (I, IV)	50	B
Pt377	*MT-ND6*	m.14439G>A:pP79S	LS	CI	100	F
Pt28	*MT-ND6*	m.14459G>A:pA72V	LS	CI	54	F
Pt593	*MT-ND6*	m.14459G>A:p.A72V	LS	CI	96	F
Pt224	*MT-ND6*	m.14487T>C:p.M63V	LS	CI	99	B
Pt1063	*MT-ND6*	m.14487T>C:p.M63V	LS	ns	Nearly 100	B
Pt396	*tRNA* ^*Glu*^	m.14687A>G	LS	CI	85	M

Pt255, identified with a mutation in *ECHS1* gene, is not listed here, and therefore the number of patients does not add up to the total number of patients with nDNA mutations on Table 1. The patient was omitted from this table because the gene analysis was processed in an outside laboratory

Segregation analyses have been completed for all autosomal recessive mutation cases

*mtDNA* mitochondrial DNA, *nDNA* nuclear DNA, *LS* Leigh syndrome, *LL* Leigh-like syndrome, *CI* isolated complex I deficiency, *CIV* isolated complex IV deficiency, *CC* combined complex deficiency, *VUS* variant of unknown significance, *ns* not significant, *na* not available, *F* fibroblasts, *M* skeletal muscle, *B* blood

^a^Pt312 is a male patient

^b^m.10197G>C is designated as VUS because the mutation confirmed in MITOMAP is m.10197G>A

Previously unreported variants were considered as potential novel causative mutations of LS/LL when they coincided with positive enzyme assay results. Mutation m.14439G>A was shown to be pathogenic using cybrid analysis (Uehara et al. 2014). One of two cases with a mutation in m.14487T>C showed a reduction in enzyme activity of complex I. Mutations m.3946G>A and m.14687A>G had been reported to cause other mitochondrial diseases (Kirby et al. 2004; Spruijt et al. 2007; Bruno et al. 2003) and were considered as causative in our patients who showed defects in respective MRC complexes. Enzyme analysis of patients with confirmed pathogenic mutations m.3697G>A, m.10158T>C, m.10191T>C, m.13513G>A, and m.14459G>A all showed defects in complex I (Kohda et al. 2016).

### nDNA analysis

Seventy-six patients proceeded to nDNA analysis, and 17 patients were identified with mutations in nine genes related to MRC complexes (*SURF1, NDUFA1*, *NDUFAF6*, *NDUFS4*, *NDUFS6, NDUFV2, BOLA3*, *SCO2*, and *GTPBP3*, see Table 4). Mutations in *NDUFAF6* and *SURF1* were most frequent (five patients each), with all patients showing reduced activity in complex I *(NDUFAF6*) or IV (*SURF1*). Mutations in genes related to complex I constituted more than half of the nDNA mutations. The genetic defects were all in agreement with the biochemical defects.

Four cases were identified with a mutation in *ECHS1*, a gene involved in valine degradation. An outside laboratory identified one more patient with a mutation in the same gene (Yamada et al. 2015). Accumulation of toxic intermediates caused by impairment in this pathway is suspected to cause MRC complex defect (Peters et al. 2014). Three of our five patients showed no decline in enzyme activities, one patient showed a defect in complex IV and another in complex I. Lastly, one patient was identified with a mutation in *SLC19A3*, a gene encoding a thiamine transporter, which is essential for cerebral thiamine metabolism.

Mutations in all these genes except *BOLA3* had been reported to cause LS (Tiranti et al. 1998; Budde et al. 2000; Fernandez-Moreira et al. 2007; McKenzie et al. 2011; Kopajtich et al. 2014; Peters et al. 2014; Gerards et al. 2013). *BOLA3* had been identified in patients with other mitochondrial diseases (Cameron et al. 2011; Haack et al. 2013), and our case was previously reported as the first evidence of this mutation in an LS patient (Kohda et al. 2016).

## Discussion

We demonstrated the importance of combining multiple methods of diagnosing LS/LL patients. Genetic analysis identified a causative mutation in 51% (53/104) of analyzed cases. Enzyme assay recognized MRC complex defects in 71% (75/106) of patients. With those approaches combined, MRC defects were confirmed in 82% (87/106) of cases. The highest diagnostic rate was reached by a combined enzymatic and genetic approach. Seven patients with normal enzyme activities had mutations in the *MT-ATP6* gene, which encodes for complex V, which is measured in few laboratories. Screening for *MT-ATP6* mutations should be performed in such settings at an early stage of diagnosis, as they comprise a significant proportion of LS/LL etiology, and screening is readily available.

Detection rates in our study of various biopsy samples were <60% individually, which confirms previous results. Most importantly, the rate in muscle biopsies was no higher than in fibroblast cell lines, a finding not reported previously. For the diagnosis of mitochondrial diseases, skeletal muscle is often considered the tissue of choice (Thorburn and Rahman 1993), and fibroblasts have been considered less sensitive than skeletal muscle biopsy samples, detecting MRC defects in only half of cases with positive skeletal muscle assay results (Thorburn et al. 2004; Heuvel et al. 2004). A similar sensitivity was observed in our study, although skeletal muscle biopsy samples returned negative results in six out of 19 cases with reduced MRC activity in fibroblasts, resulting in similar overall detection rates. Tissue specificity of mitochondrial diseases was attributed to heteroplasmy of mtDNA, but inconsistencies between materials were frequently observed in nDNA-mutated cases. These findings suggest that, when possible, more than one type of patient biological sample should be analyzed, regardless of genetic background, to improve the detection rate of mitochondrial disorder.

In pediatric practice, it can be difficult to obtain multiple biological samples, and physicians must choose selectively. Although tissues used for analysis should be taken from the most affected organ (Munnich and Rustin 2001), this is difficult to apply in principle to LS/LL, a neurodegenerative disorder of the central nervous system. So the choice would be between skeletal muscle biopsy samples and cultured fibroblast cell lines in most cases. Skeletal muscle biopsy is invasive and requires general anesthesia, which poses a risk to pediatric patients (Baertling et al. 2014). Fibroblasts, on the other hand can be obtained in office settings with local anesthesia. If only one type of material can be obtained, fibroblasts should be prioritized, as cell lines from cultured fibroblasts can be used in future studies such as those involving cybrid analysis and rescue experiments to verify the pathogenicity of novel variations (Haas et al. 2008). Should no defect be observed in fibroblasts, or if the clinical status calls for a rapid result, skeletal muscle biopsy should also be considered.

Relatively high numbers of enzyme assays return negative results in genetically verified cases of LS/LL (Sofou et al. 2014). In our study, the rate of negative assay results in genetically verified cases was 20%, excluding *MT-ATP6* mutated cases. This observation implies that a normal MRC result in muscle and/or fibroblast cell line does not exclude the possibility of a mitochondrial disorder. A reasonable proportion of MRC defects may remain undetected if negative enzyme assay results prevent us from proceeding to genetic analysis. Interestingly, negative assay results were more frequently observed in cases with nDNA than mtDNA mutations. In addition, genetic causes such as *ECHS1* mutations, which are not directly related to components of the MRC complexes, have been associated with LS/LL. In such cases, each separate MRC complex may not show reduced activity and thus remain undetected by enzyme assay. If marker substances detected by basic metabolic analysis leads directly to diagnosis, as is the case with urinary organic acids in *ECHS1* mutation, the next step is to proceed directly to analyzing the candidate gene.

In addition to genetic screening and spectrophotometric assays that measure the activity of individual respiratory complexes, we used microscale oxygraphy to help analyze mitochondrial activity. Microscale oxygraphy has a high efficiency for detecting mitochondrial respiratory defects in genetically proven mitochondrial disease patients, an observation by Invernizzi but not adopted by many diagnostic laboratories (Invernizzi et al. 2012). Half the cases in our cohort with no apparent defect in activities of MRC complexes showed a significant decline in OCR. Moreover, two nDNA mutations were identified in this group. Although evidence needs to be accumulated, this finding suggests the promising value of microscale oxygraphy as a screening tool to detect MRC defect, especially in cases in whom each complex remains intact. If cellular OCR shows a significant reduction, genetic screening should be considered, even if MRC defects were not detected by enzyme assays of fibroblasts or peripheral organs.

With advances in molecular technologies, genetic screening is becoming increasingly utilized over enzyme analysis and invasive biopsies (Lake et al. 2016; Taylor et al 2014). Enzyme assays are considered a confirmatory method for diagnosis of LS/LL in cases with ambiguous genetic results or where genetic analysis fails to detect causative mutations (Morava and Brown 2015). However, in our study, gene analysis could not identify underlying mutations in 45% of cases with reduced MRC complex activities. The genetic spectrum of LS/LL is still expanding, and biochemical data obtained via enzyme assays enable the efficient selection of candidate genes (Thorburn et al. 2004) and provide essential information in the pathogenicity of identified gene variants. Thus, enzyme analysis remains an important part of the diagnostic process of mitochondrial disorders.

Based on our increasing understanding of the biological and molecular background of the disease, new therapeutic methods are being proposed (Martinelli et al. 2012; Morava and Brown 2015). Precise biochemical and genetic diagnosis is imperative in considering the possible gene-specific therapeutic options. It is also essential to provide appropriate genetic counseling. All available biochemical and molecular methods should be combined to not only diagnose the disease but also to provide optimal care to the LS/LL patients.

## Electronic supplementary material

Table S1 (DOCX 11.4 kb).

Table S2 (DOCX 13.3 kb).
