# Supplementary material for: Clinical validity of biochemical and molecular analysis in diagnosing Leigh syndrome: a study of 106 Japanese patients
Source: J Inherit Metab Dis. 2017 Apr 20;40(5):685–93. doi: 10.1007/s10545-017-0042-6 (PMC5579154; doi:10.1007/s10545-017-0042-6)
Supplement: Supplementary file 1 — (DOCX 11.4 kb). [file 10545_2017_42_MOESM1_ESM.docx]

| Supplementary Table 1. Consistency between enzyme assay results from skeletal muscle biopsy samples and cultured fibroblasts | | | | |
| --- | --- | --- | --- | --- |
|  |  |  |  |  |
|  | | Skeletal muscle | | |
|  |  | Decreased | Normal activity | Total |
| Fibroblasts | Decreased | 13 | 6 | 19 |
|  | Normal activity | 7 | 11 | 18 |
|  | Total | 20 | 17 | 37 |
| Cases with mutations in the MT-ATP6 gene were not included in this analysis | | | | |
