# Supplementary material for: Clinical validity of biochemical and molecular analysis in diagnosing Leigh syndrome: a study of 106 Japanese patients
Source: J Inherit Metab Dis. 2017 Apr 20;40(5):685–93. doi: 10.1007/s10545-017-0042-6 (PMC5579154; doi:10.1007/s10545-017-0042-6)
Supplement: Supplementary file 2 — (DOCX 13.3 kb). [file 10545_2017_42_MOESM2_ESM.docx]

| Supplementary Table 2. Enzyme assay detection rate using cultured fibroblasts and skeletal muscle biopsy samples in genetically verified patients^*^ | | | |
| --- | --- | --- | --- |
|  |  |  |  |
|  | Fibroblasts | Skeletal muscle | Total |
| Analyzed  (nDNA/mtDNA) | 40  (23/17) | 20  (12/8) | 60  (35/25) |
| Defects detected  (nDNA/mtDNA) | 32  (17/15) | 16  (8/8) | 48  (25/23) |
| Not detected  (nDNA/mtDNA) | 8  (6/2) | 4  (4/0) | 12  (10/2) |
| Detection rate  (nDNA/mtDNA) | 80%  (74%/88%) | 80%  (67%/100%) | 80%  (71%/92%) |
| ^*^Cases with mutations in the *MT-ATP6* gene were not included in this analysis. nDNA, nuclear DNA; mtDNA, mitochondrial DNA. | | | |
